# Influence of Multimodal AR-HUD Navigation Prompt Design on Driving Behavior at F-Type-5 M Intersections

**DOI:** 10.3390/jemr19010022

**Published:** 2026-02-11

**Authors:** Ziqi Liu, Zhengxing Yang, Yifan Du

**Affiliations:** 1 School of Art and Design, Hubei University of Technology, Wuhan 430068, China; liuziqi647@hbut.edu.cn; 2 College of Design, Graduate School, Hanyang University, Seoul 04763, Republic of Korea; duyifan2203@yeah.net

**Keywords:** multimodal, AR-HUD, prompt timing, prompt mode, eye movement, F-type-5 m intersections, driving behavior

## Abstract

In complex urban traffic environments, the design of multimodal prompts in augmented reality head-up displays (AR-HUDs) plays a critical role in driving safety and operational efficiency. Despite growing interest in audiovisual navigation assistance, empirical evidence remains limited regarding when prompts should be delivered and whether visual and auditory information should remain temporally aligned. To address this gap, this study aims to examine how audiovisual prompt timing and prompt mode influence driving behavior in AR-HUD navigation systems at complex F-type-5 m intersections through a within-subject experimental design. A 2 (prompt mode: synchronized vs. asynchronous) × 3 (prompt timing: −1000 m, −600 m, −400 m) design was employed to assess driver response time, situational awareness, and eye-movement measures, including average fixation duration and fixation count. The results showed clear main effects of both prompt mode and prompt timing. Compared with asynchronous prompts, synchronized prompts consistently resulted in shorter response times, reduced visual demand, and higher situational awareness. Driving performance also improved as prompt timing shifted closer to the intersection, from −1000 m to −400 m. But no significant interaction effects were found, suggesting that prompt mode and prompt timing can be treated as relatively independent design factors. In addition, among the six experimental conditions, the −400 m synchronized condition yielded the most favorable overall performance, whereas the −1000 m asynchronous condition performed worst. These findings indicate that in time-critical and low-tolerance scenarios, such as F-type-5 m intersections, near-distance synchronized multimodal prompts should be prioritized. This study provides empirical support for optimizing prompt timing and cross-modal temporal alignment in AR-HUD systems and offers actionable implications for interface and timing design.

## 1. Introduction

Augmented Reality Head-Up Displays (AR-HUDs) are now widely used in intelligent driving assistance systems. Existing studies indicate that AR-HUDs can enhance drivers’ awareness of potential hazards while reducing overall cognitive load (i.e., mental processing demands) [1,2]. However, excessive reliance on an AR-HUD may introduce visual distraction, thereby degrading driving performance and increasing cognitive interference [3,4]. Auditory prompts offer a non-visual channel that can complement visual navigation information through multimodal integration. By distributing guidance across sensory modalities, they help reduce overload in any single channel and improve the timeliness and reliability of navigation support [5]. Importantly, the effectiveness of multimodal prompts is not determined by simple additive effects but by the temporal relationship between auditory and visual signals. Poorly timed prompts, whether presented too early or too late, may disrupt drivers’ action planning and anticipation, resulting in delayed responses or operational errors [6]. These issues are particularly evident at complex urban intersections, such as F-type-5 m intersections, where drivers are required to manage multiple concurrent demands. Under such conditions, rapid responses and effective multimodal coordination become especially critical [7]. Accordingly, a key challenge in AR-HUD interface design lies in balancing drivers’ attentional allocation between AR-HUD information and the external driving environment, with particular emphasis on optimizing auditory prompt timing.

Navigation systems typically provide information through three modes: visual-only, auditory-only, or a combination of both. Within AR-HUD navigation interfaces, visual cues remain the dominant channel. Previous studies have demonstrated that factors such as layout, icon design, and color combinations can affect drivers’ attention distribution and behavior [8,9,10]. Auditory prompts function as supplementary cues that reinforce visual information [11]. Research has shown that, compared with visual-only output, voice-based navigation can help reduce driver distraction [12]. Integrating auditory and visual modalities in multimodal navigation can further enhance driving performance and support attention management. Audiovisual combinations have been shown to accelerate braking responses and reduce collision risk [13,14]. Hu demonstrated that such integration minimizes interference with vehicle control, improving overall driving outcomes [15]. Additionally, auditory output can lower gaze scanning frequency, facilitating a more balanced distribution of attention across sensory channels [16,17]. Liu et al. [13] also highlighted that specific combinations of auditory and visual prompts improve braking performance and enhance driving safety. Collectively, these findings indicate that multimodal information output is a key approach for increasing the effectiveness and safety of AR-HUD navigation systems.

Prior research has examined visual features such as color, icons, and interface layout, as well as auditory characteristics including speech content and prompt frequency. However, relatively little attention has been given to how audiovisual prompts vary depending on timing and prompt mode. Multimodal navigation not only facilitates more effective allocation of attention at the perceptual level but also provides a framework for investigating temporal dynamics. The coordination of auditory and visual information directly affects how smoothly drivers integrate sensory cues and how well they perform. Motivated by these considerations, the current study explores how differences in audiovisual prompt timing and prompt mode influence driving behavior in AR-HUD navigation systems.

## 2. Literature Review

The timing of navigation prompts directly affects how drivers perceive and respond to navigation information [18]. Prompts presented too early can increase memory demands [6], whereas late prompts may limit decision-making time and reduce response efficiency [19]. Yun et al. [20] found that the effects of in-vehicle navigation information on lane-changing behavior vary with both traffic density and the timing of the initial cue. In AR-HUD systems, navigation information therefore needs to be delivered at moments that facilitate efficient access to task-relevant cues. Well-designed prompt timing can reduce interference and help maintain sustained driver attention [21]. Moreover, prior research suggests that auditory information typically provides stepwise guidance, while visual information offers spatial reference, and their combined effectiveness depends strongly on temporal alignment [22].

Two primary types of auditory prompts are continuous and discrete [23]. Continuous auditory prompts are typically presented in multiple, sequential stages [24]. These prompts are delivered at fixed intervals prior to a decision point (e.g., −400 m, −300 m, and −200 m), enabling drivers to maintain continuous route awareness and mitigating increases in cognitive load caused by interruptions in information flow [18,25]. However, continuous auditory prompts may lead to redundant information, fail to effectively reduce cognitive load, and even evoke negative emotional responses from drivers [26,27]. Discrete auditory prompts follow a different approach, in which a single instruction is triggered at a predefined distance (e.g., −200 m) to support the upcoming maneuver [28]. Providing auditory prompts at critical distance points enhances information clarity, improves route understanding, and reduces both cognitive load and operational uncertainty [25]. Considering AR-HUD already provides spatial guidance through the visual channel, discrete auditory prompts can supplement semantic information at critical moments. Therefore, this study focuses on the temporal coordination between auditory and visual prompts under discrete auditory prompt conditions and examines its influence on driving behavior.

In addition, the prompt modes of navigation systems can also influence driving behavior. Specifically, the synchrony or asynchrony between auditory prompts and visual information directly affects drivers’ allocation of cognitive resources [24]. Synchrony refers to the simultaneous presentation of auditory and visual prompts [29], such as when an auditory prompt saying “turn right” appears together with an AR arrow. Asynchrony, on the other hand, refers to the sequential presentation of the two modalities [29]. For example, when the AR arrow is displayed first and the auditory instruction follows with a delay, or vice versa. Studies have shown that synchronized prompts ensure information consistency and reduce the cognitive effort required to integrate cues from different sources [30,31]. At the same time, there is no clear consensus on whether strict temporal synchrony is always necessary. Chu and Huang [32] argued that humans do not depend on perfect audiovisual synchrony, and moderate asynchrony under certain conditions may not impair the accuracy of information processing. This view invites closer examination of whether strict synchrony between auditory and visual prompts is truly required in AR-HUD navigation systems. This issue becomes particularly salient when driving at multi-branch intersections or closely spaced intersections, where the prompt mode of multimodal AR-HUD guidance is especially critical. Poorly designed prompt modes may lead to driver inattention and, in severe cases, increase the risk of traffic accidents.

**Figure 1:**
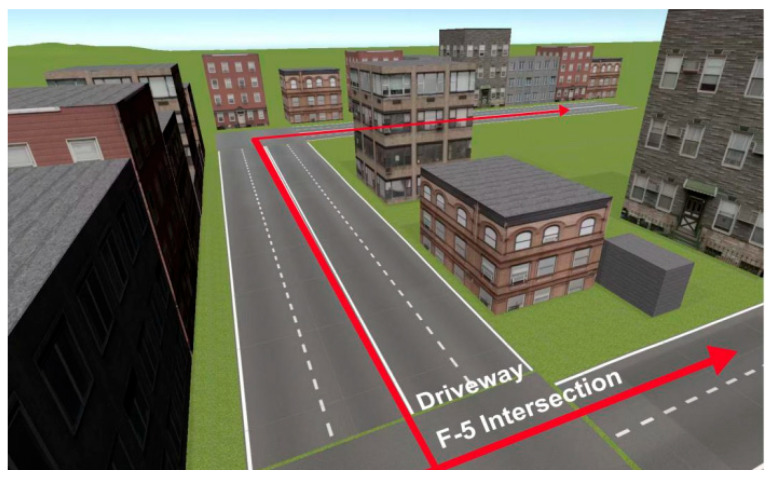
Schematic diagram of the F-type-5 m intersections.

In Chinese urban traffic environments, F-type-5 m intersections are a common but complex road structure (see Figure 1). This type of intersection includes a guiding lane that runs parallel to a cross street, with a separation distance of only five meters [18]. In this context, prompt timing and prompt mode are especially important. These intersections are usually located in high-density urban areas with heavy visual interference and frequent spatial changes, which increases the likelihood of wrong turns or missed turns during driving [33]. At intersections with multiple exits and compact spatial layouts, navigation information must be both accurate and highly time sensitive. However, few studies have specifically examined navigation prompts at F-type-5 m intersections, particularly with respect to the temporal coordination of multimodal information. Given the high cognitive demands placed on drivers in such environments, the present study adopts F-type-5 m intersections as a representative driving scenario. This study focuses on the coordination of prompt timing between discrete auditory and visual prompts in AR-HUD navigation systems and systematically investigates how synchronous and asynchronous prompt modes influence driving behavior.

Although prior studies have advanced interface design and auditory prompt optimization, the effectiveness of multimodal AR-HUD navigation is influenced by prompt timing. In addition, prompt mode, reflected in the synchronous or asynchronous coordination between auditory and visual information, also plays an important role in shaping drivers’ cognitive and behavioral responses. Moreover, empirical evidence under discrete auditory prompt conditions remains limited, especially in complex scenarios such as F-type-5 m intersections that demand precise temporal coordination. Based on this framework, the present study adopts F-type-5 m multi-exit intersections as a representative scenario and treats prompt mode (synchronous vs. asynchronous) and prompt timing (−1000 m, −600 m, −400 m) as independent variables. This study systematically examines the effects of multimodal AR-HUD navigation prompts on drivers’ situation awareness, eye-movement indicators, and reaction time under discrete auditory prompt conditions. Accordingly, two research questions are proposed.

RQ1 examines whether prompt mode of auditory and visual information in AR-HUD navigation systems significantly influences driving behavior.

RQ2 investigates which prompt timing most effectively supports multimodal integration and improves driving performance under discrete auditory prompt conditions.

For RQ1, two multimodal prompt strategies were implemented, including synchronous and asynchronous presentation. In the synchronous condition, auditory and visual prompts appeared at the same time. In the asynchronous condition, visual prompts remained continuously visible, while auditory prompts were delivered at key decision points. Behavioral reaction time, eye-movement measures (average fixation duration and fixation count), and situation awareness scores (SART) were collected to evaluate the effects of the two prompt modes on drivers’ response efficiency, attention allocation, and situation awareness. For RQ2, three prompt timing conditions were examined at −1000 m, −600 m, and −400 m. These timing points were used to compare behavioral and cognitive differences. The analysis also assessed the relative effectiveness of synchronous and asynchronous prompts at each distance.

## 3. Method

### 3.1. Participants and Apparatus

Sample size estimation was performed using G*Power 3.1.9.7 (Heinrich Heine University Düsseldorf, Düsseldorf, Germany)to ensure adequate statistical power. Previous studies indicate that an effect size of f = 0.25 corresponds to a medium effect, while values of f ≥ 0.40 reflect a large effect, both of which are considered meaningful in behavioral research [34,35]. The significance level was set at α = 0.05, and statistical power was set at 0.95 to improve the reliability of the results. Based on these parameters, the power analysis indicated a minimum requirement of 18 participants. To enhance data stability and reduce the influence of potential data loss, a total of 30 participants were recruited.

The experiment was conducted in the International Project Laboratory of the School of Art and Design, Hubei University of Technology, in a controlled laboratory setting using a fixed-base driving simulator. Participants were recruited through university-based advertisements using both online and offline channels. Prior to the experiment, participants were screened to ensure eligibility, including holding a valid driver’s license, being right-handed, and having normal or corrected-to-normal vision. Based on data quality screening, four datasets were excluded due to abnormal or incomplete experimental data, primarily resulting from incomplete task completion or technical issues affecting behavioral or eye-tracking recordings. Consequently, the final sample comprised 26 participants aged 18–26 years (M ± SD: 21.96 ± 2.34) with a balanced gender distribution. Written informed consent was obtained prior to participation, and participants received monetary compensation upon completion. The study protocol was approved by the Ethics Committee of Hubei University of Technology and complied with the Declaration of Helsinki (1975, revised 2008).

The experimental environment was developed using Unity 2022 LTS (version 2022.3.8f1), with C# scripts controlling the temporal coordination of auditory and visual prompts. The driving scene was displayed on a 34-inch high-definition monitor with a resolution of 2560 × 1440 and a viewing area of 79 × 34 cm, approximating the visual field of a vehicle windshield. A Logitech G29 driving simulator, consisting of a steering wheel, pedals, and a standard seat, was used to support realistic driving interaction. Eye-movement data were collected using Tobii Pro Glasses 3 with a sampling rate of 50 Hz, enabling real-time recording of fixation count and average fixation duration. Behavioral and eye-tracking data were synchronized and recorded on a Windows 10 laptop using Tobii Pro Lab software (version 1.208) to ensure accurate data collection for subsequent analysis.

### 3.2. Experimental Design

This study examined the joint effects of prompt timing and prompt mode on drivers’ reaction time, eye-movement behavior, and situation awareness in a multimodal AR-HUD navigation system. The experiment followed a within-subject design with a 2 (prompt mode: synchronous (SY), asynchronous (ASY) × 3 (prompt timing: −1000 m, −600 m, −400 m)) structure, resulting in six experimental conditions: −400-SY, −400-ASY, −600-SY, −600-ASY, −1000-SY, and −1000-ASY.

The experiment was conducted in a virtual driving simulation environment that represented an F-type-5 m intersection with multiple exits. Two prompt mode conditions were used. In the synchronous condition (SY), the AR-HUD visual display and auditory prompts were presented at the same time at three predefined distances (−1000 m, −600 m, and −400 m). This ensured temporal alignment between auditory and visual information. In the asynchronous condition (ASY), the AR-HUD visual display remained visible throughout the driving segment. Auditory prompts were delivered once at each of the same three distances. This setup follows common navigation practice, where visual guidance is continuously available and auditory cues are provided at key decision points. To control for order effects, the six experimental conditions were counterbalanced using a Latin square design. Each participant completed all six driving tasks under identical traffic and environmental settings. This procedure ensured consistency across conditions and supported reliable comparison of the collected data.

### 3.3. Stimulus Materials

The primary objective of this study was to define temporal coordination methods between auditory and visual prompts for multimodal prompting in AR-HUD navigation systems. The visual prompt used a gradient color scheme that transitioned from blue to red. Blue indicated a general navigation state, whereas red signaled an approaching critical point that required increased driver attention. As navigation progressed, the direction of the visual prompt was updated dynamically at each navigation node. This design reinforced spatial guidance and supported efficient recognition and attentional capture [36]. Auditory prompts were presented via headphones and consisted of natural spoken instructions. The messages provided explicit navigation guidance, for example, “In XX meters, turn right at the second intersection to enter YY Street.” This wording was selected to match the interaction style commonly used in real-world in-vehicle navigation systems.

**Figure 2:**
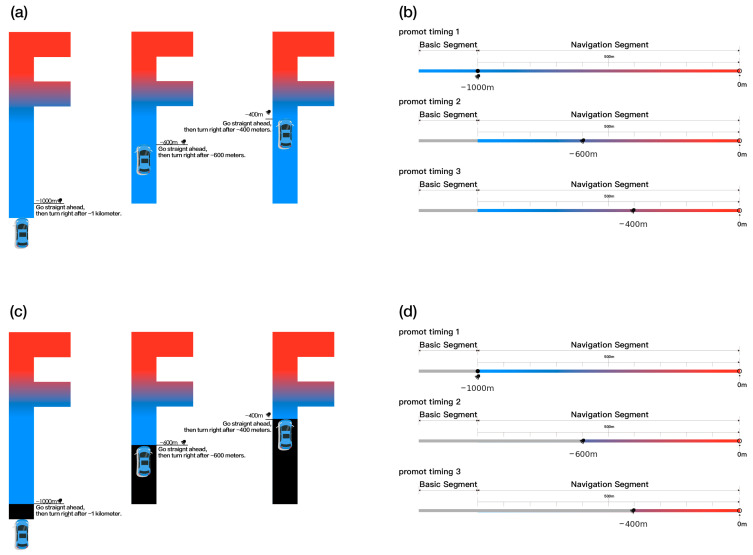
Experimental layout and prompt conditions. Panels (**a**,**b**) illustrate the asynchronous condition at three prompt timing points (−1000 m, −600 m, and −400 m). Panels (**c**,**d**) illustrate the synchronous condition at the same timing points. The color of the AR arrows indicates the proximity to the second turning intersection, with red representing closer proximity to the turning point and blue representing greater distance.

The virtual driving environment was developed in Unity 2022 LTS to provide a realistic first-person driving perspective, with the F-type-5 m intersection serving as the core experimental scenario. This intersection comprised a main roadway and a parallel guiding lane separated by a distance of 5 m, forming a characteristic F-shaped layout. Three prompt timing nodes in the experiment were predefined at approximately −1000 m, −600 m, and −400 m from the intersection. At each node, both auditory and visual navigation prompts were triggered. Two prompt mode conditions were implemented. In the synchronous mode, auditory and visual prompts were presented simultaneously at the same timing node. In the asynchronous mode, visual prompts remained continuously visible throughout the driving segment, while auditory prompts were delivered sequentially at the three timing nodes. The selection of prompt distances followed the Specifications for the Layout of Urban Road Traffic Signs and Markings (GB 5768.1-2009) [37] and corresponded to three typical driving stages, namely early anticipation, mid-course adjustment, and pre-execution. These stages reflect common driving behavior in real traffic environments and allow the effects of prompt timing to be clearly distinguished across different prompt modes. Figure 2 shows the experimental setup and the corresponding prompt conditions.

### 3.4. Procedure

Before the formal experiment, participants were provided with an informed consent form and given sufficient time to read the study objectives and procedures. Participation began only after written consent was obtained. Participants were then introduced to the experimental tasks to ensure they understood the required operations. Following the briefing, participants were seated in the driving simulator and fitted with Tobii wearable eye-tracking glasses. A standard calibration procedure was conducted before data collection. During the experiment, participants were instructed to keep their head and body movements to a minimum and to maintain a stable posture to support accurate eye-tracking recording.

**Figure 3:**
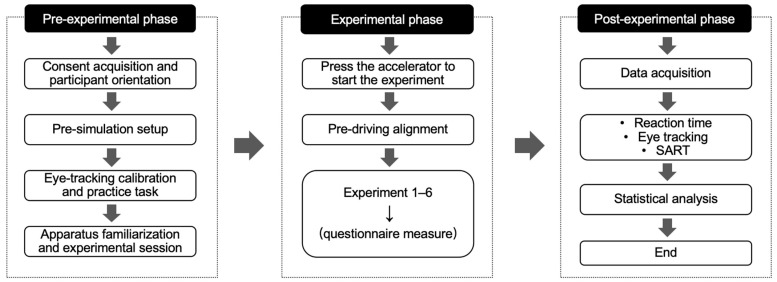
Experimental workflow. Arrows indicate progression to the next step.

Prior to the formal trials, each participant completed a short practice session to become familiar with the driving interface and task requirements. During the simulated driving trials, participants were instructed to maintain their lane, follow the speed limit, and respond to navigation instructions at the predefined prompt locations. Each participant experienced both prompt mode conditions, with three navigation prompt nodes included in each condition. To reduce external interference, dynamic traffic elements were not included, and background scenery was kept to a minimum. This allowed the effects of navigation prompts on attentional allocation to be examined under controlled conditions. After each trial, participants completed the SART questionnaire. The procedure continued until all experimental conditions were completed. An overview of the experimental procedure is shown in Figure 3.

### 3.5. Measures

To examine the effects of multimodal prompt mode and prompt timing in AR-HUD navigation on driver performance, an integrated evaluation framework was adopted. This framework combined objective behavioral measures with subjective cognitive assessments. Objective measures included reaction time and eye-movement indicators. Reaction time was measured using a simulated emergency braking task and served as an index of drivers’ response immediacy, reflecting the moment-to-moment effectiveness of navigation prompts. Eye-tracking data were analyzed to characterize gaze behavior and to infer attentional allocation and information-processing efficiency during driving.

The AR-HUD interface was defined as the Area of Interest (AOI), and two primary indices were assessed: average fixation duration (AFD) and fixation count (FC) [38]. AFD represents the time spent on each fixation within the AOI and is commonly associated with cognitive load, as inferred from eye-tracking indicators, and interface complexity. Shorter fixation durations (400–600 ms) are generally interpreted as indicating efficient information processing, whereas longer fixations (>600 ms) may reflect increased cognitive demand. FC refers to the number of fixations within the AOI. Lower fixation counts (1–3) suggest limited attentional engagement, moderate counts (4–6) indicate effective reception of visual cues, and higher counts (≥7) may signal elevated processing effort or attentional burden.

Subjective situation awareness (SA) was assessed using an extended version of the Situation Awareness Rating Technique (SART). The original SART framework proposed by Endsley (1995) [39] comprises three primary dimensions, namely supply, demand, and clarity. Following subsequent refinements to the scale [40,41,42], seven additional dimensions were included to capture information quantity, information quality, complexity, uncertainty, predictability, stress, and overall satisfaction. All items were rated on a 7-point Likert scale ranging from 1 (strongly disagree) to 7 (strongly agree). Total scores ranged from 10 to 70, with higher scores indicating greater situation awareness.

Objective measures of reaction time and eye-movement behavior were combined with subjective SART scores to form a multidimensional evaluation framework. This framework was used to examine the effects of synchronous and asynchronous prompt presentation under discrete auditory conditions on cognitive load, situation awareness, and overall driving performance.

## 4. Results

To assess the influence of prompt mode (PM: synchronous, asynchronous) and prompt timing (PT: −1000 m, −600 m, −400 m) on driving performance and cognitive processing, a two-way analysis of variance (ANOVA) was conducted. Reaction time (RT), average fixation duration (AFD), fixation count (FC), and subjective situation awareness (SA) scores were treated as dependent variables. The SA measure was derived from ten SART dimensions, including supply, demand, clarity, information quantity, information quality, complexity, uncertainty, predictability, stress, and overall satisfaction. These outcome measures were used to characterize drivers’ attentional allocation, perceptual processing, and behavioral responses across different multimodal navigation prompt conditions.

### 4.1. Reaction Time

The normality assumption was satisfied for all experimental conditions (*p* > 0.05), indicating that the data were suitable for parametric analysis. Prompt mode (PM: SY, ASY) and prompt timing (PT: −1000 m, −600 m, −400 m) were specified as independent variables, with reaction time as the dependent measure.

As reported in Table 1, a significant main effect of prompt mode on reaction time was observed (F = 20.276, *p* < 0.001, ηp^2^ = 0.119), with synchronous prompts producing shorter reaction times than asynchronous prompts. Prompt timing also showed a significant main effect (F = 12.533, *p* < 0.001, ηp^2^ = 0.143), indicating that reaction time increased as the prompt timing became later. No significant interaction effect was found between prompt mode and prompt timing (F = 0.226, *p* = 0.798, ηp^2^ = 0.003).

**Figure 4:**
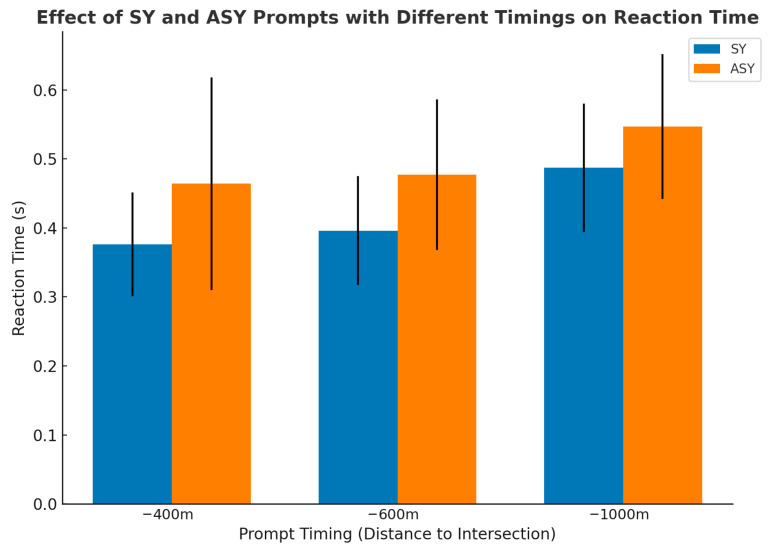
RT under SY and ASY prompts across three PT points (−1000 m, −600 m, −400 m). Error bars denote standard deviations.

Further examination of the reaction time means across conditions (see Table 2, Figure 4) showed that the shortest reaction time was observed in the −400-SY condition (M = 0.376, SD = 0.075), the fastest responses when prompts were delivered at the −400 m timing under synchronous presentation. Reaction times increased across the remaining synchronous conditions, with values of 0.396 s (SD = 0.079) for −600-SY and 0.487 s (SD = 0.093) for −1000-SY, indicating that synchronous prompts maintained relatively efficient responses across different timing points. In contrast, longer reaction times were observed under asynchronous conditions. The longest reaction time occurred in the −1000-ASY condition (M = 0.547, SD = 0.105). Reaction times in the −600-ASY (M = 0.477, SD = 0.109) and −400-ASY (M = 0.464, SD = 0.154) conditions were also higher than those recorded under the corresponding synchronous conditions.

### 4.2. Eye-Tracking Data

Eye-tracking measures were examined to assess how multimodal navigation prompts influenced drivers’ attentional allocation. The analysis focused on average fixation duration (AFD) and fixation count (FC). After confirming that the normality assumption was met, a two-way ANOVA was used to further examine the effects of prompt mode and prompt timing on eye-movement data.

#### 4.2.1. Average Fixation Duration

The ANOVA results (see Table 3) showed a significant main effect of prompt mode on average fixation duration (F = 15.915, *p* < 0.001, ηp^2^ = 0.096), with shorter fixation durations observed under the synchronous condition than under the asynchronous condition. A significant main effect of prompt timing was also found (F = 3.947, *p* = 0.021, ηp^2^ = 0.050), indicating differences in fixation duration across timing conditions. The interaction between prompt mode and prompt timing was not significant (F = 1.425, *p* = 0.244, ηp^2^ = 0.019).

**Figure 5:**
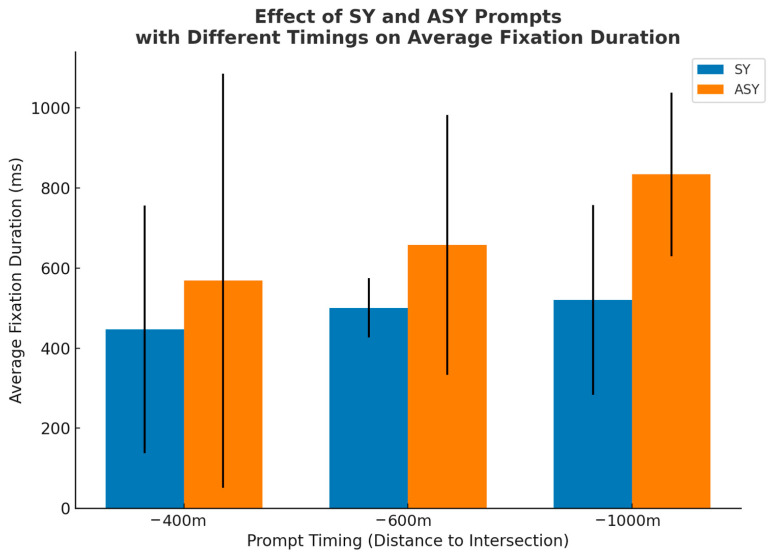
AFDs under SY and ASY prompts across three PT points (−1000 m, −600 m, −400 m). Error bars denote standard deviations.

Further examination of average fixation duration across conditions (see Table 4 and Figure 5) showed that the shortest fixation duration was observed in the −400-SY condition (M = 447.231, SD = 309.240). Short fixation durations were also found in the −600-SY (M = 500.846, SD = 74.214) and −1000-SY (M = 520.654, SD = 237.481) conditions, indicating consistently lower fixation durations under synchronous prompts across different prompt timing points. By contrast, fixation durations were generally higher under asynchronous conditions. The longest average fixation duration occurred in the −1000-ASY condition (M = 834.385, SD = 204.382). Higher fixation durations were also observed in the −600-ASY (M = 657.885, SD = 324.850) and −400-ASY (M = 568.654, SD = 517.783) conditions compared with their corresponding synchronous conditions.

#### 4.2.2. Fixation Count

Fixation count was analyzed to examine differences in visual attention across prompt conditions. The two-way ANOVA results (see Table 5) showed a significant main effect of prompt mode on fixation count (F = 6.105, *p* = 0.015, ηp^2^ = 0.039), with higher fixation counts observed under the asynchronous condition than under the synchronous condition. A significant main effect of prompt timing was also found (F = 14.674, *p* < 0.001, ηp^2^ = 0.164), indicating that fixation count increased with later prompt timing. No significant interaction was observed between prompt mode and prompt timing (F = 0.161, *p* = 0.851, ηp^2^ = 0.002).

**Figure 6:**
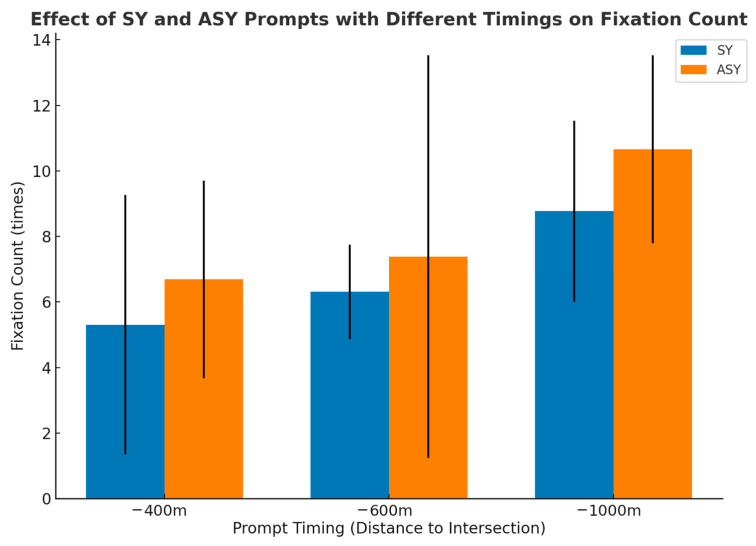
FCs under SY and ASY prompts across three PT points (−1000 m, −600 m, −400 m). Error bars denote standard deviations.

Further examination of fixation count across the six experimental conditions (see Table 6 and Figure 6) showed clear differences between conditions. The lowest fixation count was observed in the −400-SY condition (M = 5.308, SD = 3.957). Slightly higher fixation counts were recorded in the −600-SY (M = 6.308, SD = 1.436) and −1000-SY (M = 8.769, SD = 2.761) conditions, indicating relatively lower fixation counts under synchronous prompts across different prompt timing points. In contrast, fixation counts were generally higher under asynchronous conditions. The highest fixation count occurred in the −1000-ASY condition (M = 10.654, SD = 2.870). Elevated fixation counts were also observed in the −600-ASY (M = 7.385, SD = 6.145) and −400-ASY (M = 6.692, SD = 3.017) conditions compared with their corresponding synchronous conditions.

### 4.3. SART Scores

SART scores were analyzed to examine differences in drivers’ situation awareness across prompt mode and prompt timing conditions. The two-way ANOVA results (see Table 7) showed a significant main effect of prompt mode on SART scores (F = 67.483, *p* < 0.001, ηp^2^ = 0.310), with higher situation awareness scores observed under the synchronous condition than under the asynchronous condition. A significant main effect of prompt timing was also found (F = 6.804, *p* = 0.001, ηp^2^ = 0.083), indicating differences in SART scores across timing conditions. No significant interaction was observed between prompt mode and prompt timing (F = 0.385, *p* = 0.681, ηp^2^ = 0.005).

**Figure 7:**
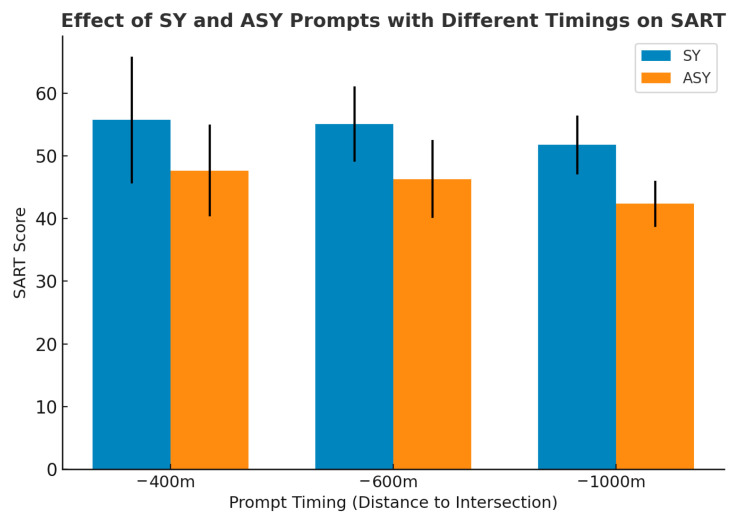
SART scores under SY and ASY prompts across three PT points (−1000 m, −600 m, −400 m). Error bars denote standard deviations.

As shown in Table 8 and Figure 7, further examination of SART scores across conditions indicated that the highest score was observed in the −400-SY condition (M = 55.731, SD = 10.102). High SART scores were also recorded in the −600-SY (M = 55.077, SD = 5.979) and −1000-SY (M = 51.731, SD = 4.678) conditions, indicating relatively high levels of situation awareness under synchronous prompts across different prompt timing points. However, SART scores were generally lower under asynchronous conditions. The lowest SART score occurred in the −1000-ASY condition (M = 42.346, SD = 3.665). Lower scores were also observed in the −600-ASY (M = 46.308, SD = 6.234) and −400-ASY (M = 47.654, SD = 7.288) conditions compared with their corresponding synchronous conditions.

To assess the reliability and validity of the SART measure, internal consistency and factor suitability analyses were performed. The scale demonstrated high internal consistency, with a Cronbach’s α of 0.915 (>0.90). Item-deletion analysis did not lead to any meaningful increase in α, supporting the stability of the scale structure. In addition, corrected item–total correlation coefficients exceeded 0.40 for all items, indicating good internal coherence across dimensions (Table 9). Construct validity was further supported by a Kaiser–Meyer–Olkin (KMO) value of 0.829 (>0.80) and a significant Bartlett’s test of sphericity (χ^2^ = 1002.511, df = 45, *p* < 0.001). These results indicate that the data were suitable for factor analysis (Table 10).

## 5. Discussion

This study investigated how prompt mode and prompt timing shape driving performance and cognitive processing in a multimodal AR-HUD navigation context. Across all dependent measures, including reaction time, eye-movement indicators, and situation awareness, a consistent pattern emerged. Synchronous prompts led to better performance than asynchronous prompts, and prompts delivered closer to the decision point were associated with more favorable outcomes. The absence of a significant interaction effect suggests that prompt mode and prompt timing contribute independently to driving performance. Rather than mutually constraining each other, these two factors appear to exert additive influences on drivers’ perceptual processing and response efficiency.

The above findings provide empirical support for RQ1 and RQ2. They demonstrate that synchronizing visual and auditory information enhances driving behavior, and that presenting navigation prompts closer to the maneuver point further strengthens perceptual efficiency and situational awareness.

### 5.1. Reaction Time Analysis

The reaction time results show that both prompt mode and prompt timing had significant effects on drivers’ response efficiency (both *p* < 0.001). Among the two factors, the effect of prompt mode was particularly robust. Synchronous presentation consistently resulted in shorter reaction times than asynchronous presentation, indicating that temporal alignment between auditory and visual cues plays a critical role in facilitating rapid driver responses. This advantage of synchronous prompting is consistent with multimodal congruency theory, which suggests that temporally aligned auditory and visual information supports faster cross-modal integration and reduces the need for additional cognitive reconciliation during response preparation [17,43]. When both modalities convey navigation information simultaneously, drivers can form a unified action representation more efficiently, leading to quicker motor responses.

Prompt timing also exerted a significant influence on reaction time (*p* < 0.001). Reaction times became shorter as the prompt timing moved closer to the decision point, indicating that information delivered later in the approach phase was more effective in supporting immediate action [44]. In F-type-5 m intersections, where multiple exits and compact spatial layouts require rapid lane selection, drivers operate under a highly constrained temporal window. If navigation cues are presented too early, their relevance may decline as a result of working-memory decay and increased cognitive maintenance demands. Similar timing effects have been reported in complex driving environments, where early prompts weaken the functional coupling between guidance information and the required maneuver, ultimately slowing responses [45].

Condition-level comparisons further illustrate these results. The shortest reaction times were observed under the −400-SY condition, suggesting that synchronous prompts delivered at a near-decision timing provide optimal support for response execution. Although reaction times increased slightly under the −600-SY and −1000-SY conditions, synchronous prompting maintained a clear performance advantage across all timing levels. In contrast, reaction times were consistently longer under asynchronous conditions, with the longest responses occurring when prompts were delivered earlier and without temporal alignment. This pattern indicates that temporal mismatch between modalities introduces additional processing costs that delay response initiation. These findings align with cognitive load accounts proposing that misalignment across information channels increases processing demands and disrupts efficient information binding [46]. When auditory and visual cues are not temporally coordinated, drivers may need to allocate extra attentional resources to resolve discrepancies, which slows response execution. By contrast, synchronous prompts reduce such overhead and support faster, more stable responses, as also observed in prior studies of multimodal navigation and warning systems [47,48].

Although no significant interaction between prompt mode and prompt timing was observed, this result is theoretically plausible under discrete auditory prompt conditions. In the present design, auditory information was delivered only once at predefined distance points, which limited continuous temporal overlap between prompt mode and prompt timing. Previous studies have shown that discrete auditory prompts primarily serve as event-based cues that support action initiation rather than sustained information processing [49]. Under such conditions, prompt mode mainly influences cross-modal integration efficiency by determining whether auditory and visual cues are temporally aligned, whereas prompt timing governs when task-relevant information becomes available relative to action execution [20,50]. Because these two factors act at different stages of cognitive processing, their effects on reaction time tend to be additive rather than interactive. Similar patterns have been reported in multimodal driving studies, where temporal synchrony improves integration efficiency, while timing proximity enhances response readiness, without producing strong interaction effects [44].

### 5.2. Eye-Tracking Data Analysis

Eye-tracking results further supported RQ1 and RQ2 by clarifying how prompt mode and prompt timing influence visual attention allocation and information processing efficiency. The analysis focused on average fixation duration and fixation count, which together reflect both the depth and frequency of visual engagement with navigation cues.

For average fixation duration, significant main effects were observed for both prompt mode (*p* < 0.001) and prompt timing (*p* = 0.021). Across all timing conditions, synchronous prompts were associated with shorter fixation durations than asynchronous prompts. Notably, AFD values in the −400-SY (M = 447.231 ms) and −600-SY (M = 500.846 ms) conditions fell within the range commonly linked to efficient visual processing, approximately 400–600 ms. This result suggests that temporally aligned audiovisual cues, particularly when delivered closer to the maneuver point, enable drivers to extract task-relevant information quickly and transition smoothly from perception to action.

In contrast, fixation durations increased markedly under asynchronous conditions. The longest AFD was observed in the −1000-ASY condition (M = 834.385 ms), substantially exceeding values recorded under synchronous prompting. This indicates that when auditory information is delayed relative to visual cues and presented at an earlier stage of the route, drivers tend to engage in extended visual dwell. Such behavior likely reflects additional verification and cross-checking processes, as drivers attempt to reconcile temporally misaligned information and reduce uncertainty regarding the upcoming maneuver. Similar effects have been reported in previous studies, which showed that temporal incongruence between modalities increases cross-modal processing load and prolongs visual engagement [43,51]. Elevated AFD values in the −600-ASY (M = 657.885 ms) and −400-ASY (M = 568.654 ms) conditions further indicate that temporal misalignment imposes additional cognitive demands even when prompts are delivered closer to the decision point [17].

A comparable result emerged for fixation count. Significant main effects were found for prompt mode (*p* = 0.015) and prompt timing (*p* < 0.001). Fixation counts were consistently lower under synchronous conditions, with the fewest fixations occurring in the −400-SY condition (M = 5.308). This finding suggests that under short-distance synchronous prompting, drivers can acquire navigation information efficiently with fewer gaze shifts, reflecting clearer cue interpretation and more stable attentional allocation. Prior research has associated reduced fixation frequency with lower information redundancy, improved interface clarity, and smoother route comprehension [2,49].

However, asynchronous prompting led to higher fixation counts across all timing conditions. The highest value was observed in the −1000-ASY condition (M = 10.654), indicating frequent attention switching and repeated confirmation behavior. This result is consistent with earlier findings showing that temporal misalignment between auditory and visual cues increases visual search demands by forcing drivers to alternate attention between HUD information and the external road environment [52]. Fixation counts in the −600-ASY (M = 7.385) and −400-ASY (M = 6.692) conditions were also noticeably higher than those in the corresponding synchronous conditions, suggesting that even at shorter distances, asynchronous prompts elevate visual search costs and require additional eye movements to resolve informational uncertainty. Overall, these results reinforce the view that temporal synchrony between auditory and visual cues promotes more efficient visual processing during navigation tasks [44].

### 5.3. SART Analysis

The SART results further demonstrate that both prompt mode and prompt timing significantly influence drivers’ situation awareness. Across all conditions, synchronous prompts yielded substantially higher SART scores than asynchronous prompts (*p* < 0.001), indicating a clearer and more stable understanding of the driving environment. This finding supports RQ1 and RQ2 and suggests that temporal alignment between audiovisual cues facilitates coherent environmental interpretation by reducing cross-modal conflict and supporting continuous situational updating. Similar effects of audiovisual synchrony on confidence, clarity, and task comprehension have been reported in previous multimodal driving studies [50,53].

Condition-level comparisons provide additional insight into how temporal structure shapes situation awareness. The highest SART score was observed under the −400-SY condition (M = 55.731, SD = 10.102), suggesting that temporally aligned multimodal prompts delivered close to the decision point are most effective in supporting drivers’ environmental comprehension and action planning. In contrast, the lowest score occurred in the −1000-ASY condition (M = 42.346, SD = 3.665). Under this condition, prompts were presented at an earlier stage and with temporal misalignment between modalities, which likely disrupted the continuity of situational information. Drivers were required to rely more heavily on memory retention and internal inference to bridge the temporal gap, thereby increasing cognitive load and reducing overall situation awareness [49]. Lower SART scores were also observed in the −600-ASY (M = 46.308) and −400-ASY (M = 47.654) conditions compared to their synchronous counterparts. This pattern indicates that even when prompts are delivered closer to the maneuver point, temporal misalignment between auditory and visual information can weaken information coherence and increase the need for verification. Prior research has similarly shown that inconsistent or fragmented information delivery increases uncertainty and hesitation, which negatively affects drivers’ ability to form a reliable understanding of road conditions [54,55].

Prompt timing itself also exerted a significant main effect on situation awareness (*p* = 0.001). As prompt distance increased, SART scores declined, suggesting that prompts delivered too early may lose relevance to the immediate driving task and impose unnecessary memory demands. Conversely, prompts delivered closer to the decision point are more tightly coupled with task requirements and therefore support more effective situational assessment. This interpretation is consistent with previous findings indicating that stronger temporal coupling between navigation information and task demands enhances situational understanding and stabilizes driving performance [44].

### 5.4. Design Implications

The present findings provide practical implications for the temporal coordination and interface design of multimodal prompts in AR-HUD navigation systems, particularly in high-decision-density environments such as F-type-5 m multi-exit intersections. Across behavioral, eye-movement, and subjective measures, synchronous prompts delivered closer to the decision point consistently supported faster responses, lower visual processing load, and higher situation awareness. These results highlight the importance of aligning multimodal information delivery with drivers’ cognitive and operational demands during complex navigation tasks.

From prompt mode perspective, AR-HUD systems should prioritize synchronous presentation of auditory and visual cues when drivers approach critical decision points. Temporal misalignment between modalities, especially under asynchronous conditions, was associated with prolonged visual verification, increased gaze shifts, and reduced situational clarity. Avoiding visual-first or audio-first delays can therefore reduce cross-modal integration costs and improve the reliability of navigation guidance in cognitively demanding traffic environments.

Prompt timing should be adjusted according to drivers’ task phases. At longer distances, such as around 1000 m, navigation systems may provide concise and low-intrusion pre-alerts to establish route direction without imposing unnecessary working-memory demands. As drivers enter the preparatory phase, approximately within the 600–400 m range, complete and synchronous audiovisual prompts become most effective. Delivering temporally aligned visual arrows together with explicit verbal instructions, such as “In 400 m, turn right at the second exit to enter YY Street,” supports action planning while minimizing visual load. Near the intersection threshold, selective visual reinforcement, for example, through increased arrow thickness, color emphasis, or route highlighting, can further support maneuver execution, whereas additional auditory input should be limited to avoid interference with immediate decision-making.

In terms of interface presentation, navigation cues should remain visually focused and low in complexity. The gradient color guidance adopted in this study proved effective in conveying directional priority and urgency without introducing excessive visual clutter. This suggests that AR-HUD interfaces may benefit from dynamic color transitions, luminance layering, and simplified arrow geometries to enhance attentional capture while preserving perceptual clarity. Key navigational elements should be positioned within the central field of view to limit gaze dispersion and reduce scanning complexity, which is particularly important in visually dense or multi-exit intersections.

In addition, the density and frequency of navigation prompts should be adaptive to roadway complexity. In exit-dense or visually congested environments, appropriately spaced prompts may help sustain situational monitoring and reduce uncertainty. In less complex road segments, lowering prompt frequency can prevent over-reliance on the navigation system and support balanced attention between interface information and the external driving environment.

Overall, these design implications emphasize that effective AR-HUD navigation in complex urban settings depends not only on what information is presented, but also on when and how it is delivered. By adopting temporally synchronized multimodal prompts and aligning their presentation with drivers’ cognitive processing stages, AR-HUD systems can reduce cognitive load, enhance situation awareness, and support safer and more reliable driver–system interaction.

### 5.5. Limitations and Future Research

Several limitations should be acknowledged. First, the participant sample consisted mainly of younger drivers, which may limit the generalizability of the findings to other driver populations. Younger drivers typically exhibit faster information processing and greater familiarity with digital navigation systems, which may influence how multimodal prompts are perceived and integrated. In contrast, previous driving simulator studies involving broader age ranges (approximately 21–55 or 21–57 years) have shown that navigation prompt timing and message structure significantly affect driving behavior and vehicle operation indicators across more representative driver populations [12,18]. While the overall direction of these findings is consistent with the present results, age-related differences in cognitive processing speed and driving strategies may moderate the magnitude of the observed effects, suggesting the need for caution when generalizing the present findings beyond younger drivers.

Second, although the simulation environment allowed precise experimental control, it could not fully capture the complexity of real-world traffic conditions. Third, prompt timing was restricted to three distance levels, and auditory prompt characteristics such as message length and semantic density were not systematically varied. These factors may influence the effectiveness of temporal coordination under different driving demands.

Future research should extend this work by including more diverse driver groups and by testing the proposed temporal strategies in on-road or higher-fidelity driving environments. Expanding prompt timing ranges, incorporating adaptive triggering mechanisms, and manipulating the informational complexity of auditory prompts may further refine multimodal navigation design. Such efforts would support the development of AR-HUD systems that better adapt to varying traffic complexity and driver needs.

## 6. Conclusions

This study investigated the effects of prompt mode and prompt timing in multimodal AR-HUD navigation systems under discrete auditory conditions, with a particular focus on complex urban scenarios such as F-type-5 m multi-exit intersections. The results demonstrate that temporal synchrony between auditory and visual prompts plays a critical role in shaping driving performance. Compared with asynchronous prompts, synchronous prompts consistently resulted in faster reaction times, more efficient visual processing, and higher situation awareness.

Prompt timing also significantly influenced performance, with prompts delivered closer to the decision point yielding more favorable outcomes across behavioral and eye-movement measures. The absence of interaction effects suggests that prompt mode and prompt timing function as independent yet complementary design dimensions. Overall, these findings provide empirical evidence for optimizing cross-modal temporal coordination in AR-HUD navigation systems to better support perception, decision-making, and driving safety in complex multi-exit intersections.

## Figures and Tables

**Table 1:** Two-way ANOVA results for RT.

Independent Variable	Dependent Variable	F	df	*p*	ηp^2^
PM	RT	20.276	1	0.000 **	0.119
PT		12.533	2	0.000 **	0.143
PM × PT		0.226	2	0.798	0.003

** *p* < 0.01.

**Table 2:** Mean RTs for each PM and PT condition.

Condition (n = 26)	−1000-SY (M ± SD)	−600-SY (M ± SD)	−400-SY (M ± SD)	−1000-ASY (M ± SD)	−600-ASY (M ± SD)	−400-ASY (M ± SD)
RT	0.487 ± 0.093	0.396 ± 0.079	0.376 ± 0.075	0.547 ± 0.105	0.477 ± 0.109	0.464 ± 0.154

**Table 3:** Two-way ANOVA results for AFD.

Independent Variable	Dependent Variable	F	df	*p*	ηp^2^
PM	AFD	15.915	1	0.000 **	0.096
PT		3.947	2	0.021 *	0.050
PM × PT		1.425	2	0.244	0.019

* *p* < 0.05, ** *p* < 0.01.

**Table 4:** Mean AFDs for each PM and PT condition.

Condition (n = 26)	−1000-SY (M ± SD)	−600-SY (M ± SD)	−400-SY (M ± SD)	−1000-ASY (M ± SD)	−600-ASY (M ± SD)	−400-ASY (M ± SD)
AFD	520.654 ± 237.481	500.846 ± 74.214	447.231 ± 309.240	834.385 ± 204.382	657.885 ± 324.850	568.654 ± 517.783

**Table 5:** Two-way ANOVA results for FC.

Independent Variable	Dependent Variable	F	df	*p*	ηp^2^
PM	FC	6.105	1	0.015 *	0.039
PT		14.674	2	0.000 **	0.164
PM × PT		0.161	2	0.851	0.002

* *p* < 0.05, ** *p* < 0.01.

**Table 6:** Mean FCs for each PM and PT condition.

Condition (n = 26)	−1000-SY (M ± SD)	−600-SY (M ± SD)	−400-SY (M ± SD)	−1000-ASY (M ± SD)	−600-ASY (M ± SD)	−400-ASY (M ± SD)
FC	8.769 ± 2.761	6.308 ± 1.436	5.308 ± 3.957	10.654 ± 2.870	7.385 ± 6.145	6.692 ± 3.017

**Table 7:** Two-way ANOVA results for SART scores.

Independent Variable	Dependent Variable	F	df	*p*	ηp^2^
PM	SART	67.483	1	0.000 **	0.310
PT		6.804	2	0.001 **	0.083
PM × PT		0.385	2	0.681	0.005

** *p* < 0.01.

**Table 8:** Mean SART scores for each PM and PT condition.

Condition (n = 26)	−1000-SY (M ± SD)	−600-SY (M ± SD)	−400-SY (M ± SD)	−1000-ASY (M ± SD)	−600-ASY (M ± SD)	−400-ASY (M ± SD)
SART	51.731 ± 4.678	55.077 ± 5.979	55.731 ± 10.102	42.346 ± 3.665	46.308 ± 6.234	47.654 ± 7.288

**Table 9:** Reliability analysis of SART.

Dimension	CITC	Cronbach’s α	Cronbach’s α
Demand	0.605	0.912	0.915
Supply	0.797	0.9	
Clarity	0.757	0.902	
Information Quantity	0.629	0.91	
Information Quality	0.733	0.904	
Complexity	0.665	0.908	
Uncertainty	0.659	0.908	
Predictability	0.682	0.907	
Stress	0.7	0.906	
Overall Satisfaction	0.666	0.908	

**Table 10:** Validity analysis of SART.

Index	Value
KMO value	0.829
Bartlett’s χ^2^	1002.511
df	45
*p*	<0.001

## Data Availability

The datasets used during the current study are available from the corresponding author upon reasonable request.

## Abbreviations

The following abbreviations are used in this manuscript:

SY	Synchronous
ASY	Asynchronous
PM	Prompt mode
PT	Prompt timing
AFD	Average fixation duration
FC	Fixation count
SART	Situation awareness rating technique
